# Challenges and solutions in communication with patients with low health literacy: Perspectives of healthcare providers

**DOI:** 10.1371/journal.pone.0267782

**Published:** 2022-05-04

**Authors:** Laxsini Murugesu, Monique Heijmans, Jany Rademakers, Mirjam P. Fransen

**Affiliations:** 1 Nivel, Netherlands Institute for Health Services Research, Utrecht, Netherlands; 2 Department of Public and Occupational Health, Amsterdam UMC Location University of Amsterdam, Amsterdam, The Netherlands; 3 Amsterdam Public Health Research Institute, Amsterdam, The Netherlands; 4 Department of Family Medicine, Care and Public Health Research Institute (CAPHRI), Maastricht University, Maastricht, The Netherlands; Dai Hoc Duy Tan, VIET NAM

## Abstract

Insights in the challenges that healthcare providers encounter in serving low health literate patients is lagging behind. This study explored challenges perceived by healthcare providers and provides strategies in communication with low health literate patients. Primary and secondary healthcare providers (*N* = 396) filled in an online survey. We assessed the frequency of challenges prior to, during and following a consultation, and which strategies were used and recommended. Survey outcomes were validated in in-depth interviews with healthcare providers (*N* = 7). Providers (76%) reported one or more challenges that were subscribed to patients’ difficulties in comprehending or applying health-related information, in communicating with professionals, or in taking responsibility for their health. Providers (31%) perceived difficulties in recognizing low health literate patients, and 50% rarely used health literacy specific materials. Providers expressed needs for support to recognize and discuss low health literacy, to adapt communication and to assess patient’s comprehension. Future research should focus on developing strategies for providers to ensure patients’ understanding (e.g. applying teach-back method), to recognize low health literate patients, and to support patients’ in taking responsibility for their health (e.g. motivational interviewing).

## 1. Introduction

Patients are expected to play active roles in decisions about their health and healthcare [1]. Not all of them are able to fulfill this role. This could be caused by several factors, such as emotional and psychological distress due to the patient’s medical status, or limited health literacy (HL) skills i.e. skills to access, comprehend, appraise and apply information to make well-informed health-related decisions [2]. Low HL skills are more likely to be seen in individuals with low socioeconomic status, immigrant background, older age or rural residence and are estimated to be present in 24,5% of the Dutch population [3, 4].

There are different types of HL skills. Osborne and colleagues have distinguished nine aspects of HL: (i) feeling understood and supported by healthcare providers, (ii) having sufficient information to manage one’s health, (iii) actively managing one’s health, (iv) social support for health, (v) appraisal of health information, (vi) ability to actively engage with providers, (vii) navigating the healthcare system, (viii) ability to find good health information and (ix) understanding health information well enough to use it [5].

Low HL may lead to unfavorable health outcomes, including increased hospitalization, greater emergency service use, poorer health status and higher mortality [6]. Patients with low HL also face challenges in different stages of their healthcare process (prior to, during, and following a consultation), such as accessing healthcare services, completing medical forms, understanding oral, written and digital health information [7, 8], or managing an illness on a day-to-day basis [9]. They often have difficulty communicating with providers, owing to poor health vocabularies, limited background knowledge and difficulties adapting to new information. Patients with low HL often report not to understand their diagnoses and treatment plans [8, 10]. Also, ineffective communication, e.g. miscommunication between provider and patients, causes poorer treatment adherence [11, 12].

Though much is known about the problems faced by low health literate patients, insight in the challenges that healthcare providers encounter in the different stages of the healthcare process and in patient communication is lagging behind. It seems that healthcare providers generally lack understanding of the prevalence and likelihood of low HL and of strategies to address it [13, 14]. Specific communication strategies, such as using simple language, providing printed materials and speaking slowly, do not seem to be routinely incorporated into clinical practice [10].

To improve healthcare provision and support healthcare providers in their communication with low health literate patients, it is essential to gain deeper insight in the challenges faced by providers, in which stages these challenges occur, and in the differences between specific provider groups [15]. This study therefore addresses the following research questions:

1. What challenges do healthcare providers experience in communicating with low health literate patients and do these differ between healthcare providers?
2. What strategies do healthcare providers use and recommend to facilitate communication?
3. What additional support do healthcare providers require to further improve communication?

## 2. Materials and methods

### 2.1. Design and sampling

This study employed a two-phase mixed methods design, where qualitative data was used to validate initial quantitative results. Using the conceptual framework explained below, we developed an online survey with closed and open-ended questions for healthcare providers. Key challenges and strategies that emerged from that survey were then discussed during in-depth interviews with healthcare providers. The purpose of the interviews was to interpret and validate the survey outcomes. Approval from an ethics committee was not required, because study participants were not subjected to actions neither were rules of behavior imposed on them.

Any Dutch primary or secondary healthcare provider consulting directly with patients was eligible for inclusion in the survey. Secondary care was defined as specialist treatment and support for patients who have been referred to specific expert care. The survey was disseminated from December 2017 to March 2018 via social media, medical association websites and an existing mailing list. One reminder was sent. Respondents identified as having exclusively management or administrative roles or unclear functions were excluded, as well as those providing only background data or less.

In-depth interviews were held by LM and MH among other healthcare providers recruited from the Dutch Healthcare Professionals Registries [16]. Healthcare providers were invited by email and asked to contact the researchers when they were interested to participate in the interviews. We used the same inclusion criteria for the interviews as for the survey, i.e. any Dutch primary or secondary healthcare provider consulting directly with patients was eligible for inclusion. Healthcare providers who participated in an interview received a gift voucher. Seven diverse healthcare providers responded to our invitation and participated in the interviews. This number was based on data saturation i.e. in the last two interviews no new information regarding interpretation of the survey was retrieved.

One interview was held by phone and the other interviews were held face-to-face at the location providers preferred.

### 2.2. Conceptual framework for the online survey of healthcare providers

**Fig 1:**
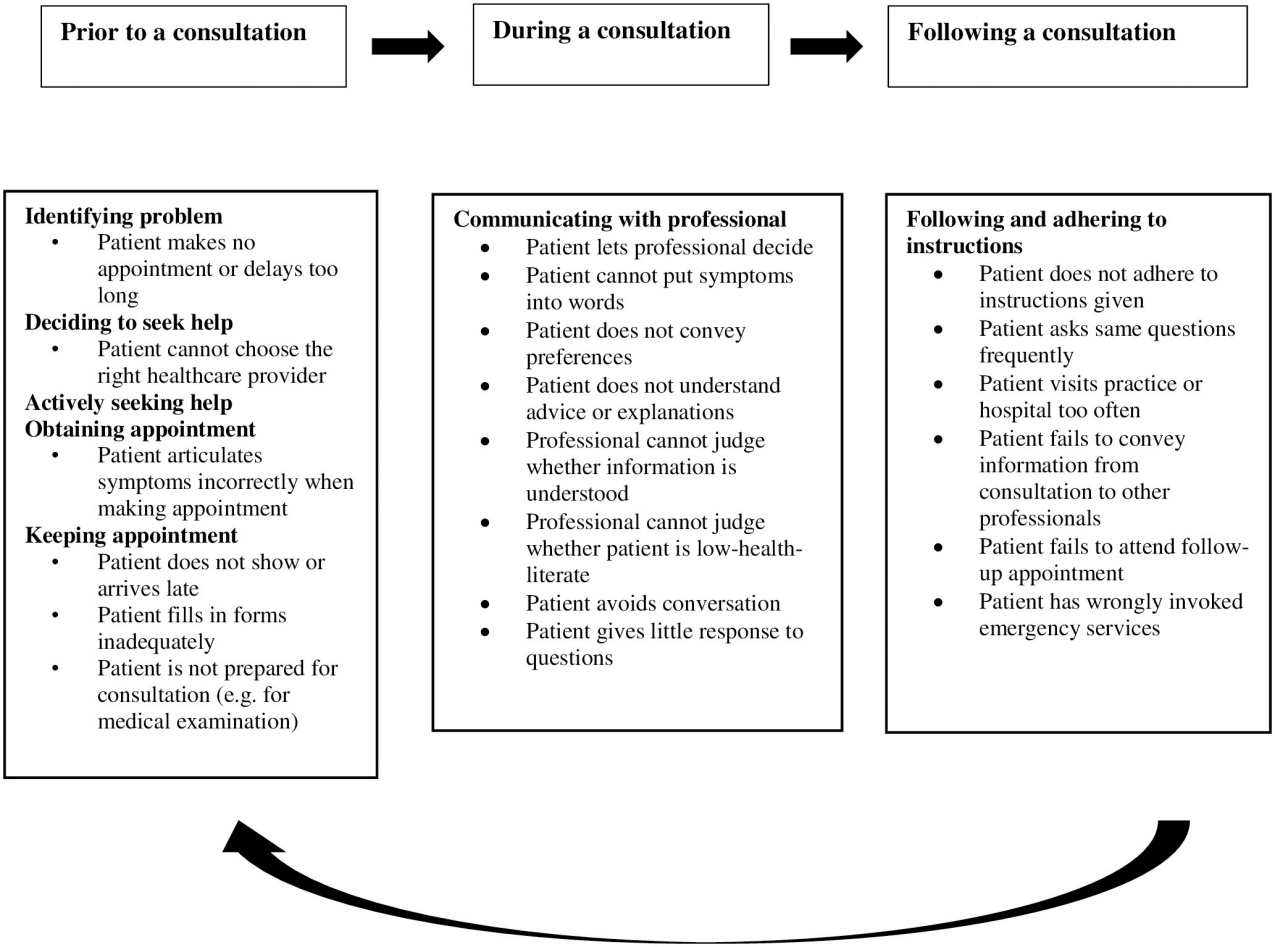
Healthcare journey in three stages with corresponding challenges*. *Conceptual framework adapted from literature [17–21].

The provider’s perspective was applied by using the Ford et al.’s model (2016). We used this framework to map challenges for a healthcare provider in communication with low health literate patients prior to, during and following a consultation. This framework was originally developed to explore the barriers that influence access to primary care for socioeconomically disadvantaged older people in rural areas. Ford et al. (2016) showed that this study population experienced personal, community and healthcare barriers that limit their access to primary care. A key mechanism underlying these challenges was low HL, which makes this framework relevant for our study. This framework explains the ‘healthcare journey’ as a flow sequence tracing the steps from a patient’s first symptoms, via medical evaluation and treatment, to living with a disease and dealing with complications. Fig 1 shows a simplified model depicting the steps in this journey (in bold) as a circular process leading to a follow-up consultation. The journey includes problem identification, decision to seek help, active search for help, obtaining and reaching an appointment, communicating with a professional, and outcome [17]. ‘Outcome’ involves the extent to which a patient adheres to the treatment plan.

Challenges prior to, during and following a consultation were tentatively predefined on the basis of literature [17–21] and then refined in a nine-member project group including communication experts, patient advocates and a policymaker. After minor adaptations, the challenges were incorporated into the framework.

### 2.3. Data collection

#### 2.3.1. Online survey of healthcare providers

The following background characteristics were assessed: type of profession, number of working days and number of years employed in current job.

The survey started with a definition of HL ‘skills that patients need to obtain, understand, appraise and use health related information to make decision about their health’ [3] (see S1 File). Experiences in communicating with low health literate patients were assessed by three multiple choice items. We explicitly asked providers to indicate how often they experienced challenges that could be attributed to low HL skills. Patient’s HL skills were operationally defined in accordance with the nine domains proposed by Osborne and colleagues [5] and the World Health Organization’s definition of HL [22]. See Table 2 for an itemization of HL skills. Based on these HL skills we assessed (1) frequency of encounters with low health literate patients (‘daily’, ‘several times weekly’, ‘once weekly’, ‘occasionally’, ‘rarely’); (2) extent to which the provider found it challenging to recognize patients with low HL (‘not challenging’, ‘moderately challenging’, ‘challenging’, ‘highly challenging’); (3) number of patients with low HL encountered weekly (‘none’, ‘fewer than 10’, ‘10 to 20’, ‘20 to 50’, ‘more than 50’, ‘don’t know’). When providers reported that they never had contact with low health literate patients, they did not receive any further questions. They only answered questions about background characteristics and strategies used to communicate with low health literate patients on organizational level. We also asked providers what additional support they would need to improve care for patients with limited HL on organizational level. Answer options were categorized in: methods or tools to improve access to care, communication and information provision.

Challenges in communicating with patients with low HL were assessed by the question *‘*How often do you experience challenges in communicating with patients with low HL?’ Predefined challenges were differentiated into three stages: prior to, during and following a consultation (Tables 3–5). Respondents could report additional challenges from their own experience in an open-ended question.

In addition to the strategies used to communicate with low health literate patients on organizational level, we also assessed the use and recommendation of predefined strategies distinguished by type of challenge (e.g. support patients to make and keep appointments) by asking:

1. Do you or your organization use methods or tools to support in ‘challenge X’?
2. If not, do you need strategies to support in ‘challenge X’?
3. If so, which methods or tools do you use? (respondents could choose predefined strategies or report other strategies that they use)
4. Do you recommend this strategy? (assessed for each strategy respondents reported to use)

Strategies that support providers in their challenges related to interaction with low health literate patients were derived from literature [6, 20, 23, 24] and previous work of the Dutch centers of expertise for Health Disparities (Pharos), Long-term Care (Vilans), and Social Development (Movisie); and a database of support tools for healthcare providers, i.e. ‘Instrumentenkiezer Zelfzorg Ondersteund!’.

#### 2.3.2. In-depth interviews with healthcare providers

The interviews with the healthcare providers started with the question ‘What challenges do you encounter in your interaction with patients with low HL?’(see S2 File). They were then asked which strategies they used to deal with each type of challenge. The questions were posed for each of the three consultation stages. Finally, we disclosed the significant challenges and commonly used strategies that had emerged in our survey and asked them to comment on these challenges and strategies and indicate whether they recognized them.

### 2.4. Data analysis

Descriptive statistics were used to summarize the background characteristics of the survey respondents, their experiences and challenges in communicating with patients with low HL, and any strategies they used and recommended to improve communication. Items measuring how frequently respondents encountered challenges prior to, during and following a consultation with low health literate patients were initially analyzed by their five-category responses. Chi-square tests were used to assess whether the four provider groups (general practice professionals, nurses, medical specialists and other healthcare providers) differed in reported challenges. Items measuring how frequently respondents encountered challenges in consulting low health literate patients were dichotomized into ‘frequently’ (‘daily’, ‘several times a week’, ‘once a week’) and ‘infrequently’ (‘occasionally’, ‘rarely’) for the chi-square tests. Differences were considered to be significant, if the p-value was less than 0.05. Quantitative analyses were conducted with STATA 15. Qualitative data consisted of the interviews and open answers in the survey. The data were coded by LM and were thematically analyzed using MAXQDA. The open questions were analyzed qualitatively; every new strategy, challenge or reason for not taking low HL into account was coded and the set of codes was used to characterize themes. The interviews with healthcare providers were audio-recorded and transcribed. The interview transcripts were summarized separately and used to supplement the survey results. Data were not double coded, but the coded text was discussed with the co-authors and themes were iteratively adapted according to project meetings with the co-authors.

## 3. Results

### 3.1. Response and background characteristics

A total of 419 healthcare providers fully or partially completed the online survey. We excluded 23 responders that had exclusively management or supporting roles, resulting in 396 inclusions. Table 1 shows the different types of professionals included in the sample. We merged general practitioners (*n* = 58) and general practice nurses (*n* = 17) into a single group of ‘general practice professionals’. The category ‘other’ mainly consisted of allied health professionals.

**Table 1:** Background characteristics survey respondents.

Background characteristics (n = 396)	n (%)	Mean (SD; range)
**Type of profession**		
General practice professionals	75 (19%)	
Nurses	179 (45%)	
Medical specialists	75 (19%)	
Other healthcare providers	67 (17%)	
**Number of years employed in current job**		17 (12; 1–45)
**Number of weekly working days average**		4 (0.9; 1–7)

The additional qualitative interviews were held with two general practitioners, two nurses, a lifestyle coach, an occupational therapist and a clinical geriatrician.

### 3.2. Experiences in communication with low HL patients

The survey revealed that 31% of the healthcare providers found it difficult to recognize patients with low HL. The majority (55%) saw at least ten low health literate patients per week in their practice.

Table 2 shows the self-reported frequency of encounters with low health literate patients, differentiated by HL skill. In total 34% to 76% of healthcare providers reported being challenged at least weekly in contacts with low health literate patients. Most reported encounters were with patients that had difficulties in understanding and applying health information, communicating effectively with providers, and in taking responsibility for own health. One in three respondents encountered patients at least weekly who have difficulty in reading and writing.

**Table 2:** Self-reported encounters with patients who have difficulties with specific HL skills (N = 396).

Patient has difficulty with…	Daily	Several times weekly	Once weekly	Occasionally	Rarely	P-value**
	(%)	(%)	(%)	(%)	(%)	
… reading and writing	8	10	16	47	19	ns
… accessing health information	12	19	30	34	6	<0.01
… understanding health information	17	22	36	21	4	<0.01
… applying health information	18	24	34	20	5	<0.01
… navigating through healthcare settings	15	19	31	29	6	<0.01
… communicating effectively with professionals	16	26	31	23	4	ns
… participating in healthcare decisions	19	23	30	24	4	<0.01
… taking responsibility for own health	21	28	27	21	4	<0.01

* Due to rounding, not all rows sum to 100%.

** p-values indicate differences in reported encounters with patients who have difficulties with specific HL skills between groups of general practice professionals, nurses, medical specialists and other healthcare providers.

In comparison with nurses, medical specialists and other healthcare providers, general practice professionals reported significantly more contacts with patients who had difficulties with specific HL skills reported in Table 2, except for the skills ‘reading and writing’ and ‘communicating effectively with professionals’.

### 3.3. Challenges in communication

#### 3.3.1. Challenges prior to a consultation

The most commonly reported challenge at this stage of the healthcare journey was no-show or arriving late. A total of 48% of respondents reported to at least weekly encounter patients who do not show or arrive late (Table 3). Some (31%) providers indicated that they weekly encounter patients who are unable to choose the right provider. General practice professionals more often deal with patients who do not show or arrive late for an appointment (*p = 0*.*04*), and patients who articulate their symptoms incorrectly (*p = 0*.*01*) than nurses, medical specialists and other healthcare providers.

**Table 3:** Frequencies in which healthcare providers encountered challenges with low health literate patients *prior to* a consultation*.

The low health literate patient…	N total	Daily (%)	Several times weekly (%)	Once weekly (%)	Occasionally (%)	Rarely (%)	P-value**
… doesn’t show or arrives late	250	8	15	25	41	11	0.04
… doesn’t make an appointment timely	250	3	15	26	43	13	ns
… inadequately completes medical or other forms	250	6	12	24	46	12	ns
… articulates symptoms incorrectly	244	6	11	24	45	14	0.01
… fails to prepare for consultation	242	4	11	22	48	15	ns
… is unable to choose the right provider	244	2	9	19	47	23	ns

* Due to rounding, not all rows sum to 100%.

** p-value indicates differences in reported challenges between groups of general practice professionals, nurses, medical specialists and other healthcare providers.

#### 3.3.2. Challenges during a consultation

Many providers indicated that they at least weekly encounter patients who leave decisions to them (59%) or who do not convey their preferences (46%) or are unable to articulate symptoms (58%). Approximately one third of respondents reported to weekly meet patients who do not understand explanations or advice (Table 4). The following challenges during a consultation were experienced more often by general practice professionals: patient leaves the decision to provider (*p<0*.*001*), does not convey preferences (*p<0*.*001*), does not understand advice (*p<0*.*001*), and does not understand explanations (*p<0*.*01*). General practice professionals also had more difficulty to gauge the HL level of patients (*p<0*.*01*) and found it more often unclear whether information is understood (*p<0*.*01*) than nurses, medical specialists and other healthcare providers.

**Table 4:** Challenges with low health literate patients *during* a consultation*.

The low health literate patient…	N total	Daily n (%)	Several times weekly n (%)	Once weekly (%)	Occasionally (%)	Rarely (%)	P-value**
… leaves decision to provider	262	10	19	30	35	7	<0.001
… is unable to articulate symptoms	272	8	17	31	40	4	ns
… doesn’t convey preferences	264	6	13	27	44	10	<0.001
… doesn’t understand advice	267	4	6	23	59	8	<0.001
… doesn’t understand explanations	269	4	7	22	59	8	<0.01
… avoids conversation	263	2	7	18	51	23	ns
… gives little response to questions	265	4	4	15	45	32	ns
Provider is unclear as to whether information is understood	271	4	8	20	57	11	<0.01
Provider has difficulty to gauge as to HL level	265	3	8	16	60	13	<0.01

* Due to rounding, not all rows sum to 100%.

** p-value indicates differences in reported challenges between groups of general practice professionals, nurses, medical specialists and other healthcare providers.

In the open-ended survey questions, providers also highlighted a lack of knowledge in some patients about their own health and lifestyle as well as an inability to appraise non-validated information (e.g. from the Internet). Additional challenges involved dealing with patient’s anger, aggression or fears during a consultation.

#### 3.3.3. Challenges following a consultation

More than half of the healthcare providers (56%) reported encountering patients at least weekly who do not adhere to instructions (Table 5). General practice professionals dealt significantly more often with patients not adhering to instructions, contacting their provider more than necessary, not attending follow-up appointments, and wrongly invoking emergency services than nurses, medical specialists and other healthcare providers.

**Table 5:** Challenges with low health literate patients *following* a consultation*.

The low health literate patient…	N total	Daily (%)	Several times weekly (%)	Once weekly (%)	Occasionally (%)	Rarely (%)	P-value**
… doesn’t adhere to instructions	261	9	15	32	37	7	<0.001
… asks same questions frequently	264	4	9	30	47	9	ns
… contacts provider more than necessary	242	5	10	22	45	19	<0.001
… fails to convey information from consultation to other providers	242	7	5	25	54	10	ns
… doesn’t attend follow-up appointment	235	1	3	15	58	23	<0.001
… has wrongly invoked emergency services	235	3	5	8	47	37	<0.001

* Due to rounding, not all rows sum to 100%.

** p-value indicates differences in reported challenges between groups of general practice professionals, nurses, medical specialists and other healthcare providers.

### 3.4. Strategies

#### 3.4.1. Healthcare provider and organizational sensitivity to patients’ HL skills

Our survey revealed that healthcare providers sometimes (33%) or never (8%) took low HL into account when communicating with low health literate patients orally or in writing. Time constraints were mentioned as the primary reason for not taking low HL into account. Other reasons given by various providers were a lack of financial resources; a lack of clarity about the HL level; assumptions that others had already adequately compensated for HL (e.g. through numbered routes in hospitals), therefore addressing HL related challenges was not seen as providers’ responsibility; assumptions that patients are sufficiently informed in advance via e-mail or leaflets; and insufficient awareness about low HL.

Half of the surveyed healthcare providers mentioned that they or the organization in which they are employed sometimes (41%) or never (9%) made systematic use of HL-specific materials. The most-cited reasons were: unfamiliarity with the notion of low HL; lacking awareness of the available adapted materials; inadequate knowledge of the nature and extent of low HL, and hence of the urgent need to adjust; unavailability of suitable materials within the organization; and the assumption that a patient has received the required information before the consultation.

#### 3.4.2. Strategies used and recommended to improve care to low health literate patients

Table 6 presents frequently reported strategies that were used and recommended to other healthcare providers, distinguished by type of challenge. The first column presents the denominator, i.e. the frequency of providers who reported to use strategies to deal with a specific challenge. The second column shows the percentages of healthcare providers who recommend the strategy that they reported to use. Frequently used and recommended strategies were tailored communication and strategies to determine whether a patient has understood information, including using visualization tools, and repeating and summarizing information. The teach-back method was recommended by 99% of those using it. This is a strategy whereby providers ask patients to paraphrase the information in their own words to ensure they have understood it correctly [25]. Other strategies that were used and recommended by over 90% of the respondents, were helpful attitudes throughout staff, e.g. help filling out medical forms, responding to patients’ ideas, concerns and expectations, and motivational interviewing.

**Table 6:** Used and recommended strategies, differentiated by type of challenge.

Strategies by type of challenge		
	Providers using strategies to deal with this challenge (N total, %)	Providers using and recommending this strategy (N, %)
**Support in making and keeping appointment**	201 (100%)	
• Sending e-mail or text reminders or providing appointment slips		179 (89%)
• Scheduling at easy-to-remember times (e.g. on the hour or half hour)		54 (27%)
• Linking appointments to a daily activity (e.g. ‘before you start work’)		36 (18%)
**Support in articulating problems or needs**	117 (100%)	
• Encouraging bringing a companion to the consultation		96 (82%)
• Being readily approachable for making appointments		77 (66%)
• Encouraging patients to make lists of questions beforehand		59 (50%)
**Support in completing medical forms**	78 (100%)	
• Helpful attitudes throughout staff		70 (90%)
• Forms available in multiple languages		40 (51%)
**Support in navigating through hospital or GP practice**	123 (100%)	
• Volunteers to help patients navigate		89 (72%)
• Lucid information about hospital or practice		79 (64%)
• Short video or other materials to explain routes in hospital or practice		25 (20%)
**Making low HL negotiable**	103 (100%)	
• Responding to patients’ ideas, concerns and expectations		94 (91%)
• Establishing patients’ preferences for their own role in decision-making		61 (59%)
• Displaying posters in waiting rooms, about issues such as low literacy		36 (35%)
**Supporting staff in recognizing low HL**	54 (100%)	
• Staff information sheets on recognizing low HL		26 (48%)
• Tricks such as handing patients leaflets upside down while discussing them		23 (43%)
**Adapting communication and information materials to patients’ HL levels**	133 (100%)	
• Repeating and summarizing information		125 (94%)
• Using visualization tools (videos, pictures, drawings)		122 (92%)
• Avoiding medical terminology		114 (86%)
• Using short sentences in active voice		114 (86%)
**Assessing whether patient has understood information**	162 (100%)	
• Teach-back method		160 (99%)
**Supporting shared decision-making**	140 (100%)	
• Using decision aids to discuss treatment options		74 (53%)
• Using the Ask 3 Questions approach to improving health communication by encouraging patients to ask three questions during each visit: What are my options? What are the potential benefits and risks? How can we make a decision together that is right for me?		55 (39%)
**Motivating patient to plan behavioral change**	130 (100%)	
• Motivational interviewing		124 (95%)

Additional strategies cited by healthcare providers in the open-answer section among others were more consultation time with low health literate patients, call patient to remind them of the appointment, e-learning modules, and simply worded take-home information.

### 3.5. Reported needs for strategies

**Fig 2:**
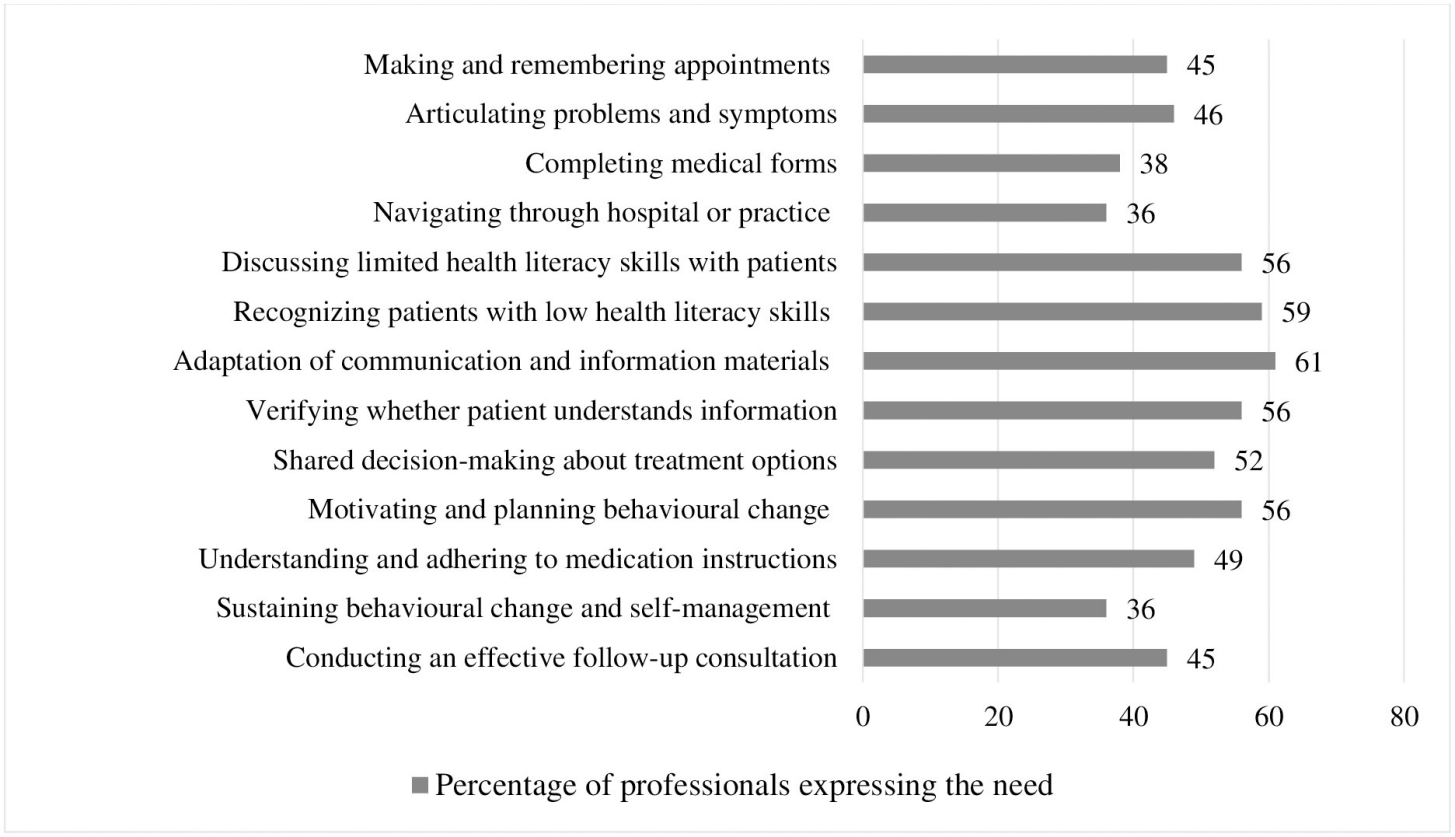
Percentages of professionals expressing needs for low health literate support strategies, by type of challenge (responders *n* = 70–205).

Fig 2 gives an overview of providers who reported not to use strategies for each challenge presented, and mentioned that they do need strategies to support their patients (in percentages). Providers especially reported to need strategies to adapt communication and information materials, to recognize and discuss low HL skills in practice, to check whether the patient has understood information and to motivate the patient to plan behavioral change.

### 3.6. Qualitative interviews with healthcare providers

#### 3.6.1. Challenges prior to consultation

In the interviews providers reported being more alert to possible low HL skills if patients had an intellectual disability, came from low-SES neighborhoods, were elderly or spoke poor Dutch. They emphasized that low HL was less recognizable in other types of patients, who may hide their illiteracy, hesitate to convey failure to understand, or give socially desirable answers. As the occupational therapist for example mentioned *“I actually never had people [patients] who could not read or write*. *Or I wouldn’t know right*? *Because they are probably very good at hiding it*.*”*

#### 3.6.2. Challenges during a consultation

General practitioner 1 explained during the interview, that she encounters patients who cannot clearly convey their symptoms, explaining: “*I find it extremely difficult to say*, *I don’t understand what you are saying*, *or can you repeat what you’ve said*?”. Interviewees also pointed to a tendency among professionals to keep conversations medical and general–possibly difficult for low health literate patients to follow. Furthermore, interviewees confirmed that they encounter patients who cannot choose the right provider, noting further that many presenting care needs were non-medical in nature, including complex social problems such as financial difficulties, family circumstances and housing. The occupational therapist stated: “*Sometimes I tell them to consult the municipality for Social Support*, *but they find it difficult to contact them*. *So you have to do more work*, *than what you are supposed to do as an occupational therapist*.*”*

#### 3.6.3. Challenges after a consultation

Interviewed healthcare providers observed that some patients with low HL make repeated appointments and need more reassurance than others. As the general practitioner (2) mentioned, *“This type of patients worry a lot*, *therefore you have to explain everything to reassure them*.*”* Taking responsibility for one’s own health was not seen by providers as a challenge specific to low health literate individuals. Taking responsibility could be difficult for people with adequate HL as well, and it could be linked to poor habits, motivation and self-confidence. *“I am not sure if they don’t understand it [referring to advices about physical activity] or if they don’t want to do it*. *Some people deliberately choose to make certain physical movements*. *That is linked to their motivation*.*”*–Occupational therapist.

## 4. Discussion

### 4.1. Main findings

In total, 76% of the primary and secondary healthcare providers in this survey were confronted at least weekly with one or more challenges in their contacts with patients who have low levels of HL. About 75% of the providers weekly encountered patients who had difficulty comprehending and/or applying health information, taking responsibility for their health, or with whom communication proceeded difficultly. General practice staff experienced significantly more challenges than other professionals with patients who do not show up to (follow-up) appointments, who have difficulties articulating their symptoms and preferences, who leave the decision to the provider, and who do not understand and follow medical instructions. In addition, they dealt significantly more often with patients who repeatedly visit their practice or wrongly invoke emergency services. Finally, general practice staff had more difficulty to gauge the HL level of patients and were more often unclear as to whether information is understood. However, taking responsibility for one’s own health was not seen by interviewees as a challenge specific to low health literate individuals. Taking responsibility could be difficult for people with adequate HL as well, and it could be linked to poor habits, motivation and self-confidence.

Interviewees further noted that many presenting care needs were non-medical in nature, including complex social problems such as financial difficulties, family circumstances and housing. They reported being more alert to possible low HL skills when patients had an intellectual disability, were elderly or spoke poor Dutch. Still, providers emphasized that low HL was less recognizable in other types of patients, who may hide their illiteracy, hesitate to convey failures to understand, or give socially desirable answers. Half of the providers never, or only sometimes, adapted their communication or used HL-specific materials. This was largely attributable to low professional awareness of the notion of HL and of the strategies available to address HL challenges, to difficulties recognizing low HL and to perceived shortage of time. Frequently used strategies were responding to patients’ ideas, concerns and expectations, repeating and summarizing information, using visualization tools, applying the teach-back method and motivational interviewing. The teach-back method was the most-recommended strategy.

Providers reported being especially in need of strategies and techniques to recognize and discuss low HL, to adapt communication materials and to judge the comprehension capabilities of low health literate patients.

#### 4.1.1. Discussion of main findings

Challenges mentioned by providers were in line with the definition of HL, i.e. skills to access, comprehend, appraise and apply information to make well-informed health-related decisions. Providers highlighted a lack of knowledge in some patients about their own health and lifestyle as well as an inability to appraise non-validated information (e.g. from the Internet). The inability to appraise non-validated information could be especially problematic for patients who do not make an appointment (timely), because they may do their own information searching instead. Searching for online health information requires digital HL skills, i.e. “the ability to seek, find, understand, and appraise health information from electronic sources and apply the knowledge gained to addressing or solving a health problem” [26]. Low health literate patients are similarly vulnerable to have low digital HL skills [27].

Two frequently highlighted challenges in our survey were patients’ lack of participation in decision-making and their difficulties in explaining symptoms, problems or needs–skills that are necessary components of shared decision-making. Another study also showed that low HL can be a barrier to implementing any decisions made [28]. Healthcare providers in other studies stressed that patients with low HL may lack a range of skills needed for decision-making [29]. To avoid inequity, that is implementing shared decision-making only among those who are equipped to participate, the decision-making process should be recommended for all patients, but tailored to individuals’ abilities [30]. Patients with at least one chronic illness also mention that providers should be flexible in their behavior and communication depending on the particular patient [31].

Another barrier for providers in practicing shared decision-making, which is also confirmed by other studies is time constraints. Our study showed that shortage of time was a major barrier for providers to adapt communication techniques or apply HL-specific strategies to support patients in tasks like decision-making. Investing more time to explain complex information could be assumed to leave less time for shared decision-making. However, a systematic review on the subject has found no robust evidence that shared decision-making requires more time than routine clinical practice [30].

Another frequently reported challenge was taking responsibility for one’s own health, which is a key construct of HL [32]. Studies have shown that low health literate patients may have difficulties understanding medication instructions, appointment reminders, informed consent procedures, hospital discharge instructions and health education materials [25]. Consequently they may show poorer adherence to medication regimens, skip appointments and lab tests, and perform health self-management less adequately [25]. We also found that general practice staff more often dealt with patients who wrongly invoke emergency care services and repeatedly visit their practices. However, Vandenbosch et al. (2016) found no significant relation between patients’ HL level and the number of GP or emergency consultations. They did find that low health literate patients have significantly more hospitalizations and more GP visits at home than patients with adequate HL [33].

Our study showed that providers, especially general practice staff, experienced difficulty with recognizing low health literate patients. Other studies have shown that professionals often miss cues that would reveal low HL skills in patients, and that patients may be adept at concealing such deficiencies [34]. Storms et al. (2019) showed that general practitioners were often unable to estimate HL levels of their patients. The HL levels in patients identified with inadequate HL, were considerably overestimated by their general practitioners [35].

The teach-back method was the most recommended strategy by healthcare providers in our study. In the past decade healthcare providers are increasingly encouraged to apply the teach-back method, since this method ensures that providers are constantly checking patients’ comprehension in a non-blaming way [36]. Research has found it to be associated with better health outcomes [25]. However, a systematic review has found that the teach-back method has been mostly used so far as a pilot intervention rather than a routine practice component [37]. This could explain the discrepancy in our survey between the broad-scale recommendation of this strategy and the rates of its actual use. Hence, providers need encouragement to develop the habit of routinely applying the teach-back method [36].

#### 4.1.2. Strengths and limitations

A strength of our study is its mixed-methods design, allowing us to quantitatively investigate challenges and strategies, while still remaining open to new perspectives [38].

A limitation of this study is that we did not pretest the survey to assess clarity of the questions. Another limitation is that healthcare providers who participated in the survey may have had a heightened interest in improving HL. The results may therefore portray a best-case scenario for communication behaviors in healthcare. They may underestimate the needs for further training and education. Moreover, as the online survey was disseminated through several different media, we could not calculate a response rate or gauge the representativeness of the sample. Our finding that 34% to 76% of the surveyed healthcare providers encountered various challenges in their communication with patients with low HL may well be an underestimation, given their broad-scale acknowledgement of their difficulties in recognizing low HL.

### 4.2. Practice implications

This study explores challenges, strategies and needs of healthcare providers in their communication with patients with low HL. The most common reported challenges were related to patients’ difficulty comprehending and/or applying health information, taking responsibility for their health, or patients with whom communication proceeded difficultly. Healthcare providers reported strategies to tackle these challenges. For example, to support patients in comprehending and/or applying health information strategies such as the teach-back method, visualizing, summarizing and repeating information could be used. Providers could apply motivational interviewing to support patients in taking responsibility for their health and illness. Reasons not to apply strategies were low professional awareness of the notion of HL, difficulties recognizing low HL and perceived shortage of time. Our study showed that strategies that were less often used, but might be helpful to recognize patients with low HL are for example displaying posters in waiting rooms, about issues such as low literacy, and training staff members in recognizing low HL. This is especially important for general practice staff considering their gatekeeping role in many healthcare systems.

Our study results could support researchers, policymakers and educators in developing strategies tailored to the needs and challenges of healthcare providers and patients, and in training providers in communicating with low health literate patients. The systematic development of HL-specific strategies in diverse healthcare disciplines could substantially improve providers’ skills in communicating with patients with low HL. Improving healthcare providers’ skills in communicating with low health literate patients is one of the factors that could contribute to more active roles among patients. In addition, besides low HL there are also other factors on patient-level that could hinder participation and should be taken into account, such as psychological and emotional distress. Also, active participation among patients cannot be fully achieved without taking other factors into account including organizational health literacy, i.e. the degree to which healthcare organizations implement strategies to make it easier for patients to understand health information, navigate the health care system, engage in the health care process, and manage their health [39].

## 5. Conclusion

In total, 76% of the healthcare providers in this survey faced one or more challenges in their contacts with low health literate patients. The challenges were significantly more often experienced by general practice staff than by nurses, medical specialists and other providers. Despite this, a significant proportion did not systematically adapt their communication or used materials to accommodate their patients’ lack of HL skills. Providers expressed needs for further strategies to recognize and discuss low HL skills in practice settings. Future research can support the systematic development of dedicated communication strategies to overcome low HL.

## Supporting information

S1 File Online survey–Communication with low health literate patients. (DOCX)

S2 File (DOCX)
