# Supplementary material for: Challenges and solutions in communication with patients with low health literacy: Perspectives of healthcare providers
Source: PLoS One. 2022 May 4;17(5):e0267782. doi: 10.1371/journal.pone.0267782 (PMC9067671; doi:10.1371/journal.pone.0267782)
Supplement: S1 File — (DOCX) [file pone.0267782.s001.docx]

**Supporting information 1. Online survey – Communication with low health literate patients**

1. **Please indicate your background below.**

Organization and department: ………………………………………………………………………..………………………………………………

Profession:
❑ general practitioner
❑ general practice nurse

❑ nurse
❑ medical specialist
❑ other………..

How many days a week do you work? .........................................................................................................

How long have you been employed? (in years)

................................................................................................................

**Patients with low health literacy**

This questionnaire is about the care for patients with limited health literacy skills. Health literacy skills are skills that patients need to obtain, understand, assess, and use health information to make decisions about their health and healthcare.

1. **Please indicate how often you encounter patients who have difficulty with one or more of these skills.**

| To what extent do you encounter patients in your daily work who have difficulties with ... | **Daily** | **Several times weekly** | **Once weekly** | **Occasionally** | **Rarely** |
| --- | --- | --- | --- | --- | --- |
| ... reading and writing | ❑ | ❑ | ❑ | ❑ | ❑ |
| ... accessing health information | ❑ | ❑ | ❑ | ❑ | ❑ |
| ... understanding health information | ❑ | ❑ | ❑ | ❑ | ❑ |
| ... applying health information | ❑ | ❑ | ❑ | ❑ | ❑ |
| ... navigating through healthcare settings | ❑ | ❑ | ❑ | ❑ | ❑ |
| ... communicating effectively with professionals | ❑ | ❑ | ❑ | ❑ | ❑ |
| ... participating in healthcare decisions | ❑ | ❑ | ❑ | ❑ | ❑ |
| ... taking responsibility for own health | ❑ | ❑ | ❑ | ❑ | ❑ |

1. **How difficult / easy is it for you to recognize patients with limited health literacy skills?**

❑ very easy

❑ somewhat easy

❑ somewhat difficult

❑ very difficult

1. **How many patients with limited health literacy skills do you encounter at work (for example during consultation hours)? (keep an average work week in mind)**

❑ none – continue with question 7

❑ very few (<10)

❑ some ( 10 – 20)

❑ many (20 – 50)

❑ very many (> 50)

❑ I don’t know

**Challenges from your perspective as a healthcare provider in communicating with patients with low health literacy skills**

Below we present a list of challenges you may encounter in your contact with or care for patients with limited health literacy skills.

1. For each challenge that you think is related to limited health literacy skills, please indicate how often you have to deal with this yourself?

| **Challenges prior to a consultation** | **Daily** | **Several times weekly** | **Once weekly** | **Occasionally** | **Rarely** |
| --- | --- | --- | --- | --- | --- |
| **The patient doesn’t show or arrives late** | ❑ | ❑ | ❑ | ❑ | ❑ |
| **The patient doesn’t make an appointment timely** | ❑ | ❑ | ❑ | ❑ | ❑ |
| **The patient inadequately completes medical or other forms** | ❑ | ❑ | ❑ | ❑ | ❑ |
| **The patient articulates symptoms incorrectly** | ❑ | ❑ | ❑ | ❑ | ❑ |
| **The patient fails to prepare for consultation** | ❑ | ❑ | ❑ | ❑ | ❑ |
| **The patient is unable to choose the right provider** | ❑ | ❑ | ❑ | ❑ | ❑ |

| **Challenges during a consultation** | **Daily** | **Several times weekly** | **Once weekly** | **Occasionally** | **Rarely** |
| --- | --- | --- | --- | --- | --- |
| **The patient leaves decision to provider** | ❑ | ❑ | ❑ | ❑ | ❑ |
| **The patient is unable to articulate symptoms** | ❑ | ❑ | ❑ | ❑ | ❑ |
| **The patient doesn’t convey preferences** | ❑ | ❑ | ❑ | ❑ | ❑ |
| **The patient doesn’t understand advice** | ❑ | ❑ | ❑ | ❑ | ❑ |
| **The patient doesn’t understand explanations** | ❑ | ❑ | ❑ | ❑ | ❑ |
| **The patient avoids conversation** | ❑ | ❑ | ❑ | ❑ | ❑ |
| **The patient gives little response to questions** | ❑ | ❑ | ❑ | ❑ | ❑ |
| **Provider is unclear as to whether information is understood** | ❑ | ❑ | ❑ | ❑ | ❑ |
| **Provider has difficulty to gauge as to HL level** | ❑ | ❑ | ❑ | ❑ | ❑ |

| **Challenges following a consultation** | **Daily** | **Several times weekly** | **Once weekly** | **Occasionally** | **Rarely** |
| --- | --- | --- | --- | --- | --- |
| **The patient doesn’t adhere to instructions** | ❑ | ❑ | ❑ | ❑ | ❑ |
| **The patient asks same questions frequently** | ❑ | ❑ | ❑ | ❑ | ❑ |
| **The patient contacts provider more than necessary** | ❑ | ❑ | ❑ | ❑ | ❑ |
| **The patient fails to convey information from consultation to other providers** | ❑ | ❑ | ❑ | ❑ | ❑ |
| **The patient doesn’t attend follow-up appointment** | ❑ | ❑ | ❑ | ❑ | ❑ |
| **The patient has wrongly invoked emergency services** | ❑ | ❑ | ❑ | ❑ | ❑ |

1. Do you encounter any other challenges in your communication with patients who you believe have limited health literacy skills?

❑ no

❑ yes
 Could you indicate what other challenges you encounter in communicating with patients who you believe have limited health literacy skills?

1. ………………………………………………………………………………
2. ………………………………………………………………………………
3. ………………………………………………………………………………

**Solutions, methods and tools**

To what extent do you, or the organization in which you work, take patients with low levels of health literacy into account? You may take into account limited health literacy skills in your communication, information, or education. The questions below address this topic.

1. Do you, or the organization in which you work, take patients’ limited health literacy skills into account when communicating with them, verbally or in writing?

❑ no

Could you please explain why you or the organization do not take patients’ limited health literacy skills into account when you communicate with them, verbally or in writing? ………………………………………………………………………………

❑ yes, sometimes

❑ yes, often

❑ yes, always

Can you indicate which method(s) you or the organization use when taking patients’ health literacy levels into account when you communicate with them, orally or in writing?

1. ………………………………………………………………………………
2. ………………………………………………………………………………
3. ………………………………………………………………………………
4. Do you, or the organization in which you work, take patients’ limited health literacy skills into account when providing information (such as websites, leaflets, information letters)?

❑ no

Could you please explain why you or the organization do not take patients’ limited health literacy skills when providing information (such as websites, leaflets, information letters)?
………………………………………………………………………………

❑ yes, sometimes

❑ yes, often

❑ yes, always

Can you indicate which method(s) you or the organization use when taking patients’ health literacy into account when providing information (such as websites, leaflets, information letters)?

1. ………………………………………………………………………………
2. ………………………………………………………………………………
3. ………………………………………………………………………………
4. What (additional) methods or tools do you need to improve care for patients with limited health literacy? (multiple answers possible)

- methods or tools to improve access to care
- methods or tools to improve communication
- methods or tools to improve information (oral, written, online)
- other ………………………………………………………………………………
- none, I don't need any methods or tools

1. We ask you to identify any tools or methods you use and would recommend to other healthcare providers in your communication with patients with limited health literacy skills. If you don’t use any tools or methods to address the specific challenge, we ask you to indicate whether you would like to use it.

We will then ask you to select the three most useful tools for you.

|  |  | |  | | |
| --- | --- | --- | --- | --- | --- |
|  |  | | **Would you recommend this strategy?** | | |
| Do you or your organization use methods or tools to … | yes | no | yes | no | not sure |
| **Support in making and keeping appointments** | ❑ | ❑ | ❑ | ❑ | ❑ |
|  | If so, which methods or tools do you use? (multiple answers possible)   - Sending e-mail or text reminders or providing appointment slips - Scheduling at easy-to-remember times (e.g. on the hour or half hour) - Linking appointments to a daily activity (e.g. ‘before you start work’)   ❑ other………………. | | | | |
|  | If not: Do you need strategies to support in making and keeping appointments?   - Yes - No - Not sure | | | | |
| **Support in articulating problems or needs** | ❑ | ❑ | ❑ | ❑ | ❑ |
|  | If so, which methods or tools do you use? (multiple answers possible)   - Encouraging bringing a companion to the consultation - Being readily approachable for making appointments - Encouraging patients to make lists of questions beforehand   ❑ other………………. | | | | |
|  | If not: Do you need strategies to support in articulating problems or needs?   - Yes - No - Not sure | | | | |
| **Support in completing medical forms** | ❑ | ❑ | ❑ | ❑ | ❑ |
|  | If so, which methods or tools do you use? (multiple answers possible)   - Helpful attitudes throughout staff - Forms available in multiple languages   ❑ other………………. | | | | |
|  | If not: Do you need strategies to support in completing medical forms?   - Yes - No - Not sure | | | | |
| **Support in navigating through hospital or GP practice** | ❑ | ❑ | ❑ | ❑ | ❑ |
|  | If so, which methods or tools do you use? (multiple answers possible)   - Volunteers to help patients navigate - Lucid information about hospital or practice - Short video or other materials to explain routes in hospital or practice   ❑ other………………. | | | | |
|  | If not: Do you need strategies to support in navigating through hospital or GP practice?   - Yes - No - Not sure | | | | |
| **Make low health literacy negotiable** | ❑ | ❑ | ❑ | ❑ | ❑ |
|  | If so, which methods or tools do you use? (multiple answers possible)   - Responding to patients’ ideas, concerns and expectations - Establishing patients’ preferences for their own role in decision-making - Displaying posters in waiting rooms, about issues such as low literacy   ❑ other………………. | | | | |
|  | If not: Do you need strategies to make low health literacy negotiable?   - Yes - No - Not sure | | | | |
| **Support staff in recognizing low HL** | ❑ | ❑ | ❑ | ❑ | ❑ |
|  | If so, which methods or tools do you use? (multiple answers possible)   - Staff information sheets on recognizing low HL - Tricks such as handing patients leaflets upside down while discussing them   ❑ other………………. | | | | |
|  | If not: Do you need strategies to support staff in recognizing low HL?   - Yes - No - Not sure | | | | |
| **Adapt communication and information materials to patients’ HL levels** | ❑ | ❑ | ❑ | ❑ | ❑ |
|  | If so, which methods or tools do you use? (multiple answers possible)   - Repeating and summarizing information - Employing visualization tools (videos, pictures, drawings) - Avoiding medical terminology - Using short sentences in active voice   ❑ other………………. | | | | |
|  | If not: Do you need strategies to adapt communication and information materials to patients’ HL levels?   - Yes - No - Not sure | | | | |
| **Assess whether the patient has understood information** | ❑ | ❑ | ❑ | ❑ | ❑ |
|  | If so, which methods or tools do you use? (multiple answers possible)   - Teach-back method   ❑ other………………. | | | | |
|  | If not: Do you need strategies to assess whether the patient has understood information?   - Yes - No - Not sure | | | | |
| **Support shared decision-making** | ❑ | ❑ | ❑ | ❑ | ❑ |
|  | If so, which methods or tools do you use? (multiple answers possible)   - Using decision aids to discuss treatment options - Employing the Ask 3 Questions approach to improving health communication by encouraging patients to ask three questions during each visit: What are my options? What are the potential benefits and risks? How can we make a decision together that is right for me?   ❑ other………………. | | | | |
|  | If not: Do you need strategies to support in shared decision-making?   - Yes - No - Not sure | | | | |
| **Motivate patients to plan behavioral change** | ❑ | ❑ | ❑ | ❑ | ❑ |
|  | If so, which methods or tools do you use? (multiple answers possible)   - Motivational interviewing   ❑ other………………. | | | | |
|  | If not:  Do you need strategies to motivate patients to plan behavioral change?  • Yes  • No  • Not sure | | | | |

1. If you had to recommend methods or tools to other healthcare providers to improve their communication with patients with limited health literacy, what would your top 3 be?
2. ………………………………………………………………………………
3. ………………………………………………………………………………
4. ………………………………………………………………………………

Could you explain your choice?

………………………………………………………………………………

1. Thank you for your cooperation. If you have any questions or comments, you can leave them below.

|  |
| --- |
