# Supplementary material for: Challenges and solutions in communication with patients with low health literacy: Perspectives of healthcare providers
Source: PLoS One. 2022 May 4;17(5):e0267782. doi: 10.1371/journal.pone.0267782 (PMC9067671; doi:10.1371/journal.pone.0267782)
Supplement: S2 File — (DOCX) [file pone.0267782.s002.docx]

- Explain definition of health literacy: skills to access, comprehend, appraise and apply information to make well-informed health-related decisions
- How do you recognize low health literate patients?
- What challenges do you encounter in your communication with low health literate patients? (prior to, during, and following a consultation)
- What strategies do you use to deal with these challenge? (prior to, during, and following a consultation)
- Present top 4 challenges from survey
  - The patient doesn’t show or arrives late (or arrives too early)
  - The patient leaves decision to provider
  - The patient is unable to articulate symptoms
  - The patient doesn’t adhere to instructions
- Do you recognize these challenges? How do you deal with these challenges?
- Present top 4 strategies from survey
  - Support in making and keeping appointment
  - Assessing whether patient has understood information
  - Support shared decision-making
  - Adapt communication and information materials to patients’ health literacy levels
- Do you use these strategies?
- What additional support do you need the most when addressing challenges in your communication with low health literate patients?
